# Go Where the Virus Is: An HIV Micro-epidemic Control Approach to Stop HIV Transmission

**DOI:** 10.9745/GHSP-D-19-00418

**Published:** 2020-12-23

**Authors:** Michael M. Cassell, Rose Wilcher, Reshmie A. Ramautarsing, Nittaya Phanuphak, Timothy D. Mastro

**Affiliations:** a FHI 360, Hanoi, Vietnam.; b FHI 360, Durham, NC, USA.; c Institute of HIV Research and Innovation, Bangkok, Thailand.; d Center of Excellence in Transgender Health, Chulalongkorn University, Bangkok, Thailand.

## Abstract

Essentially all HIV transmission is from people living with HIV who are not virally suppressed. An HIV micro-epidemic control approach that differentiates treatment support and prevention services for people living with HIV and their network members according to viral burden could optimize the impact of epidemic control efforts.

## Key Messages

- Essentially all HIV transmission is from people living with HIV (PLHIV) who do not know their infection status or have not yet achieved viral suppression, making support for these individuals and their risk contacts a priority for treatment and prevention efforts.
- Proven approaches exist to reduce viral burden and interrupt HIV transmission from PLHIV who are not yet virally suppressed, but these approaches must be implemented with enhanced focus and scale to maximize benefit.
- Improved diagnostic approaches offer new opportunities to increase public health impact by prioritizing support for unserved or underserved individuals with the greatest viral burdens and among members of their risk networks.
- Policy makers should pursue the implementation and evaluation of diagnostic approaches that can focus services among individuals and networks with the greatest viral burdens.
- Program managers should treat viral burden as a primary consideration in the provision of differentiated HIV services, applying an HIV micro-epidemic control framework to prioritize and tailor services for PLHIV and their risk contacts along a continuum of progression to viral suppression.

## INTRODUCTION

Globally, a growing majority (59%) of an estimated 38 million people living with HIV (PLHIV) know their HIV status and have achieved HIV viral suppression by adhering to antiretroviral therapy (ART).^1^ Individuals who achieve sustained viral suppression and undetectable levels of circulating virus through good adherence to ART live long, healthy lives and will not transmit HIV through sexual contact.^2^ ^–^ ^4^ The evidence that people who have achieved undetectable viral loads will not transmit HIV sexually—that “undetectable equals untransmittable” (U=U)—underscores the prevention benefits of treatment and the rationale for the global call to achieve near-universal access to ART and viral suppression among PLHIV.^4^ ^–^ ^6^

Conversely, HIV viral burden (viremia), generally measured by plasma viral load (HIV RNA copies/mL) assays, is the primary predictor of HIV-related disease progression, morbidity, mortality, and ongoing transmission.^4^ ^,^ ^7^ Essentially all HIV transmission originates from a shrinking minority of PLHIV globally (41%) who do not know their HIV infection status or have not yet achieved viral suppression,^8^ making support for these individuals and their risk contacts a priority for treatment and prevention efforts. Studies have identified a dose-response relationship in which each 10-fold increase in HIV plasma viral load results in an increased relative risk of HIV transmission of 2.5 to 2.9 per sexual contact.^9^ ^,^ ^10^ Emerging evidence suggests that even under conditions of near-universal HIV treatment coverage, high viremia and high levels of risk behavior among unserved or underserved PLHIV can sustain epidemic HIV transmission.^11^ ^,^ ^12^ In a recent U.S. study of HIV patients in care with a detectable viral load, only a small proportion of PLHIV reported concurrent sexual transmission risk behaviors, but most of the individuals in this group had considerably elevated viral loads, increasing the probability of transmission. The study found that viral loads were likely to be lower among those with a detectable viral load who reported always using condoms.^13^

High viral burden associated with acute HIV infection (AHI) is a particular concern. Acute infection is characterized by a 2–4-week period of exceptionally high viremia as HIV replicates rapidly in the body before a person’s immune system mounts a response and reduces the level of circulating virus to much lower—but typically not undetectable—levels for a period of months to years.^14^ ^,^ ^15^ Although only a small proportion of all PLHIV will be in this brief AHI phase at any given time, per-sex-act transmission probabilities are considerably higher during periods of acute as compared to chronic HIV infection.^9^ ^,^ ^16^ ^–^ ^18^

In key populations engaged in frequent behavioral risks, up to an estimated 50% of all HIV transmission occurs from individuals during AHI when viremia is very high prior to the development of an immune response including anti-HIV antibodies (Ab) that yield reactivity on third-generation Ab assays.^14^ ^,^ ^17^ ^,^ ^19^ ^–^ ^24^ The provision of ART during AHI and of HIV pre-exposure prophylaxis (PrEP) to the risk-network contacts of acutely infected individuals could prevent a substantial proportion of ongoing HIV transmission. An analysis in Thailand suggested that early diagnosis and treatment during AHI among men who have sex with men could avert 89% of all new infections in this population.^25^

Approaches that differentiate service delivery to better address the preferences and needs of unserved and underserved individuals have been identified as a priority to close outstanding gaps in access to HIV prevention and treatment.^26^ In implementing differentiated services, it is increasingly clear that a focus on individuals and networks with the greatest viral burdens has strategic benefit. For example, programs typically transition individuals who are receiving HIV treatment and are identified through routine viral load testing as virally suppressed to options for less frequent clinical follow-up and multimonth dispensing of their antiretroviral medications. This differentiation offers additional convenience to patients and frees up resources and provider time to focus support on virally unsuppressed individuals with greater adherence, clinical, social support, and other needs.

**Figure:**
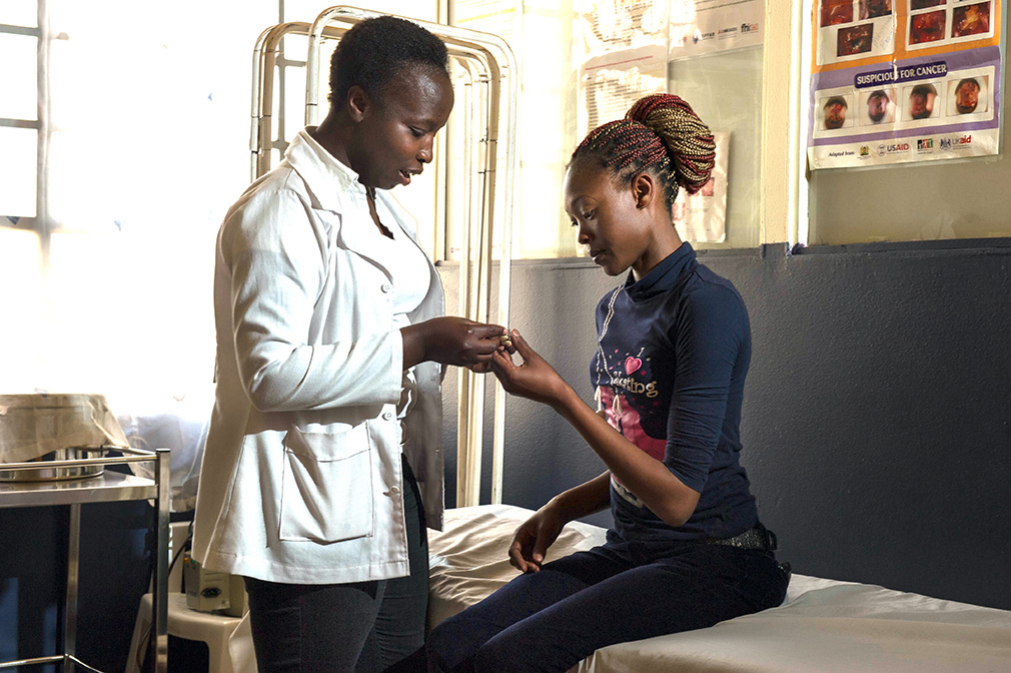
Liz Brenda Kandeyi, nurse (left), takes a client through the steps for clinical services at Kikuyu Sasa Center, Nairobi, Kenya. © 2017 Nancy Coste/FHI 360

Nevertheless, the resources and technologies needed to activate a more comprehensive differentiation of support based on viral burden historically have been limited. With the advent of expanded access to viral load testing and options to screen for AHI, opportunities now exist to prioritize support for individuals and in risk networks with the greatest viral burdens. This prioritization can help interrupt epidemic HIV transmission associated with AHI through early diagnosis, HIV treatment, and provision of PrEP and other proven prevention approaches to risk contacts. Because HIV morbidity, mortality, and transmission risk are most closely associated with viral burden, this enhanced focus can guide the allocation of limited resources to maximize the impact of prevention and treatment efforts.

Prioritizing viral load testing and screening options for individuals with the greatest viral burdens can help interrupt epidemic HIV transmission.

## ENVISIONING A MICRO-EPIDEMIC CONTROL APPROACH THAT DIFFERENTIATES SUPPORT BASED ON VIRAL BURDEN

We propose an HIV micro-epidemic control framework to characterize these opportunities to accelerate impact, with a primary focus on addressing the differentiated service preferences and needs of individuals who are not yet virally suppressed, as well as the members of their risk networks. This framework aims to organize and integrate both new and existing approaches to tailor support for PLHIV and their risk contacts based on progression to sustained viral suppression. By profiling the characteristics of those who face challenges in achieving viral suppression—such as barriers to diagnosis, to treatment initiation and retention post-diagnosis, and to viral load testing and suppression—programs can introduce solutions that both help these individuals and that remove barriers for others.

By profiling the characteristics of those who face challenges in achieving viral suppression, programs can introduce solutions that both help these individuals and remove barriers for others.

**FIGURE 1.**
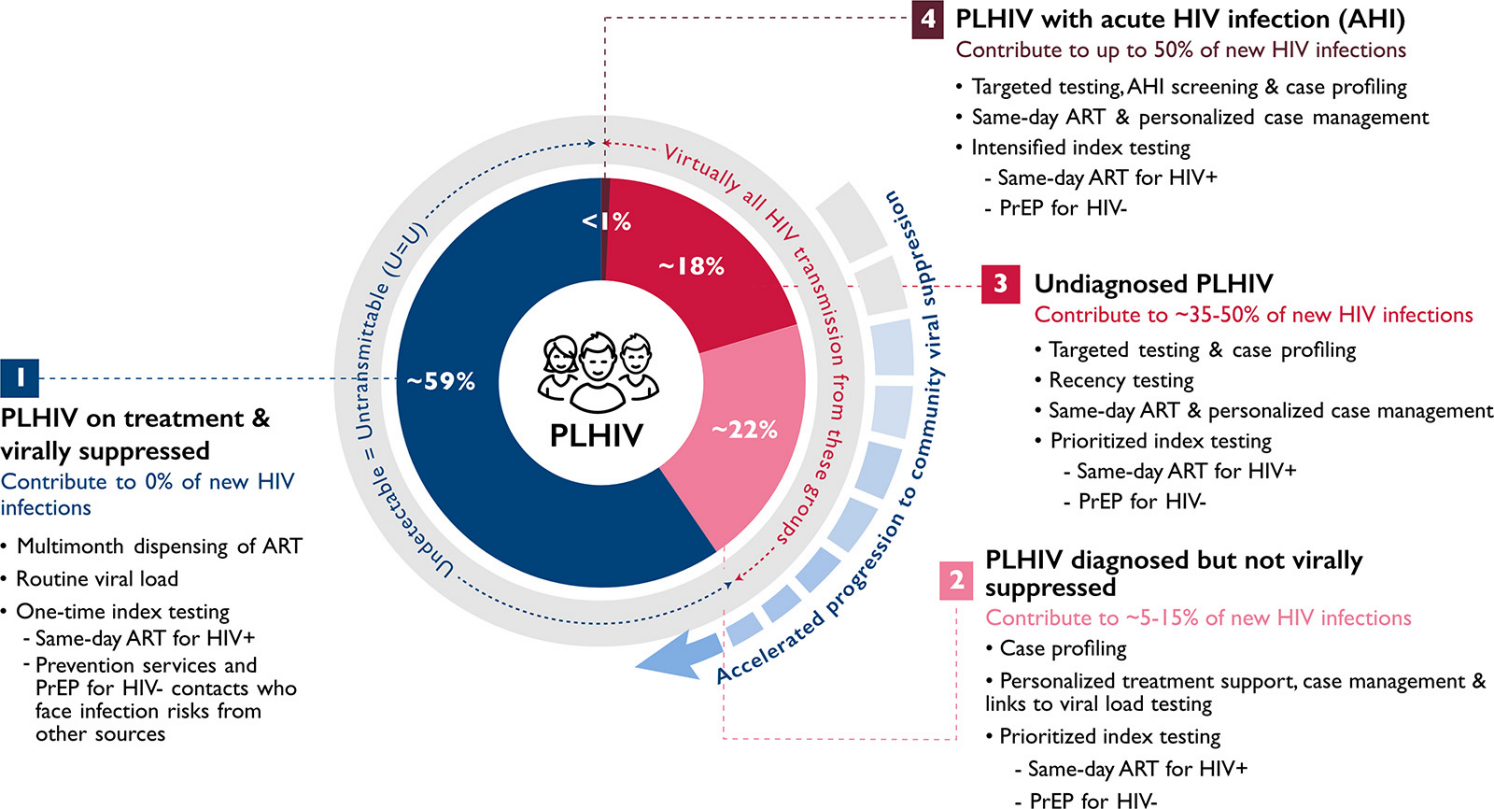
An HIV Micro-epidemic Control Model Aims to Prioritize and Focus Treatment and Prevention Efforts Where They Can Have the Greatest Impacts: Among a Shrinking Proportion of Individuals and Risk Networks With the Greatest Viral Burdens Abbreviations: AHI, acute HIV infection; ART, antiretroviral therapy; HIV+, HIV-positive; HIV-, HIV-negative; PLHIV, people living with HIV; PrEP, pre-exposure prophylaxis.

The approach features variable treatment and prevention services and service intensity grouped according to 4 different stages along this continuum of progression to viral suppression (Figure 1). The model also affords program managers with opportunities to prioritize program efforts based on regional, national, and subnational variations in progress with respect to the expansion of HIV prevention, testing, treatment, and viral suppression coverage.

### 1. PLHIV Who Are on Treatment and Virally Suppressed

Although progress varies by region, an estimated 59% of PLHIV globally are already receiving HIV treatment and are virally suppressed.^8^ Importantly, per “U=U,” these individuals will not transmit HIV to their sexual partners.^4^ PLHIV who are found to be virally suppressed through routine viral load testing are good candidates for multimonth dispensing, which will reduce the need for more frequent clinic visits, a benefit for both PLHIV and clinic staff.

Partner notification services, also known as index testing, are recommended by the World Health Organization as a safe, effective strategy to accelerate HIV epidemic control by asking PLHIV to list and refer their sexual and injecting partners and biological children to HIV testing services on a voluntary basis.^27^ Offering index testing at least once to these individuals can help link members of their networks who may previously have been exposed to HIV to relevant testing, prevention, and treatment services. Uninfected network members will not acquire HIV infection from sexual contact with PLHIV who have undetectable viral loads, but those who continue to be at elevated HIV infection risk from other contacts can be offered PrEP and other HIV prevention services, including condom education and access. Routine viral load testing is critical to monitor and sustain viral suppression among PLHIV who have previously achieved suppression. To facilitate viral load testing access and the provision of efficient retention and adherence support to PLHIV with a stable treatment status, programs can implement virtual online- and telephone-based support with patient consent and appropriate measures in place to ensure the security and confidentiality of patient information. The use of point-of-care viral load testing technologies may also reduce testing turnaround times and bring added convenience to patients and providers.

### 2. PLHIV Who Are Diagnosed but not Virally Suppressed

A substantial proportion of individuals who have previously received an HIV diagnosis have either not yet initiated ART or have not achieved viral suppression.^8^ Individuals in this group can be further divided into 3 categories: (1) those who have never been linked to ART; (2) those who have initiated ART but have not yet achieved viral suppression or have been lost to follow-up and stopped ART; and, (3) those who are sustained on treatment but are showing signs of breakthrough viremia or treatment failure. For individuals who have never initiated treatment or been lost to follow-up, programs can initiate outreach campaigns through clinical or community staff to engage or reengage previously diagnosed individuals. These campaigns can promote “U=U” messaging, the benefits of new dolutegravir-based treatment regimens,^28^ and convenient and confidential options for PLHIV to access same-day HIV treatment. For individuals who are receiving treatment but have not achieved viral suppression, providers can offer additional personalized adherence counseling and support. Immediate support should be provided to individuals with a viral load test indicating an unsuppressed viral load to help to identify and address root causes of adherence or treatment failure that require regimen switching.

Immediate support should be provided to PLHIV who are diagnosed but not virally suppressed to help address root causes of adherence or treatment failure.

In the process of providing personalized treatment support to PLHIV, a range of voluntary index testing options can be offered to encourage referrals of their risk contacts to prevention services such as PrEP and condoms until they have achieved viral suppression, or to HIV treatment services, as needed.^27^ It may also be useful for programs to routinely monitor the sociodemographic and risk characteristics of individuals who do not initiate treatment, fall out of care, or do not achieve viral suppression, to assess how these individuals differ from those who engage in treatment and sustain good treatment outcomes. By generating profiles of the characteristics of individuals more likely to face treatment challenges, programs can apply these to prevent loss and other adverse outcomes, helping to accelerate and sustain progression to viral suppression. Some programs are applying machine learning algorithms to automate this process of preventive prioritization to enhance care.^29^

### 3. Undiagnosed PLHIV

PLHIV who have not yet received a diagnosis can be similarly offered tailored support to maximize individual treatment and population-level prevention benefits. An expanded range of options for accessing HIV testing services, including HIV self-testing options with dispensing through pharmacies and peer networks, can help to close gaps in diagnosis among PLHIV who might otherwise not otherwise access diagnostic or other services.^27^ ^,^ ^30^ ^,^ ^31^ Testing services also can be tailored to focus on key populations facing the greatest HIV infection risks and to engage the risk network members of PLHIV who are not virally suppressed. Incorporating AHI screening into these targeted testing approaches can improve the detection of AHI among individuals who might otherwise have remained undiagnosed. While offering voluntary index testing and risk contact referral services to individuals who are newly diagnosed or who have recent HIV infection as identified though recency testing, programs can also assess the differentiating sociodemographic and risk profiles of these individuals. These profiles can be applied to further enhance the focus of HIV testing efforts by bringing testing to individuals with similar profiles and by engaging peer mobilizers with similar characteristics to make testing referrals and distribute HIV self-testing kits in their networks.

### 4. PLHIV With AHI

Expanding screening for AHI among key populations and other individuals facing elevated HIV infection risks can help realize the largely untapped treatment and prevention benefits of identifying, treating, and index testing individuals with AHI. To maximize efficiency, AHI screening can be prioritized for the risk network members of individuals identified with AHI, those with recent HIV infections, and newly identified PLHIV. Screening can also be focused on key populations reporting recent behavioral risks, as well as among those with other sexually transmitted infections. Upon diagnosis, all PLHIV can be offered an accelerated path to viral suppression with same-day treatment initiation. Programs can assess the differentiated characteristics of newly diagnosed, recently infected, and acutely infected PLHIV to further optimize the relevance and focus of HIV testing efforts.

While the treatment and treatment-as-prevention benefits of prioritizing support according to viral burden may have evident advantages, the HIV micro-epidemic control approach also aims to enhance prevention benefit by focusing services in the risk networks in which active HIV transmission is occurring. The majority of risk contacts of PLHIV who are undiagnosed will be uninfected but at high risk of acquiring HIV infection, making linkages of these individuals to prevention services a priority. These focused prevention efforts should employ a combination of evidence-based prevention strategies relevant to the specific preferences and needs of the populations being served.^32^ ^,^ ^33^ These strategies include, but are not limited to, harm reduction programming for people who inject drugs, support for correct and consistent condom use, and expanded access to PrEP. To maximize uptake, services should be implemented in a friendly manner that is welcoming and convenient to clients and is responsive to their feedback.

The HIV micro-epidemic control approach aims to enhance prevention benefit by focusing interventions in the risk networks in which active HIV transmission is occurring.

For all risk contacts of PLHIV who are not yet virally suppressed, PrEP is a critical, evidence-based, and likely short-duration priority.^34^ Making PrEP—and, as relevant, nonoccupational HIV postexposure prophylaxis—offers routine for the contacts of PLHIV as part of index testing affords enormous opportunities to focus PrEP where it can have the greatest prevention impact. In circumstances where partners and other risk contacts face no other substantial HIV infection risks, these individuals can safely discontinue PrEP once the PLHIV index client has achieved viral suppression. In addition, the scale up of PrEP as part of index testing services may serve to normalize PrEP and expand availability across a wider array of settings, helping to accelerate historically limited progress towards the achievement of global PrEP targets^35^ and removing barriers to access among men who have sex with men and individuals who may prefer not to disclose their status as key populations.^36^

Stigma, discrimination, violence, and other structural factors impose considerable barriers to service uptake, particularly among PLHIV and key populations living in criminalizing environments.^37^ ^,^ ^38^ Therefore, the micro-epidemic control approach should be implemented in conjunction with broader efforts to address these structural factors. By identifying the characteristics of individuals for whom structural factors serve as particular barriers to health and well-being, a micro-epidemic control framework may help bring additional focus to structural interventions and facilitate advocacy and partnerships with individuals and communities to develop and implement voluntary, safe, equitable, and preferred policy and program solutions.

## THE CHALLENGE OF ACUTE HIV INFECTION

The proposed micro-epidemic control approach emphasizes diagnosis of and intervention during AHI in light of the substantial role that AHI plays in epidemic transmission of HIV. Most current national HIV testing algorithms rely on antibody-based serological testing that cannot detect AHI. As a result, these approaches misdiagnose potential core transmitters as HIV-uninfected and miss critical opportunities to maximize the prevention benefits of HIV treatment. Affordable, accurate, and scalable solutions to diagnose AHI have remained elusive.^17^

**FIGURE 2.**
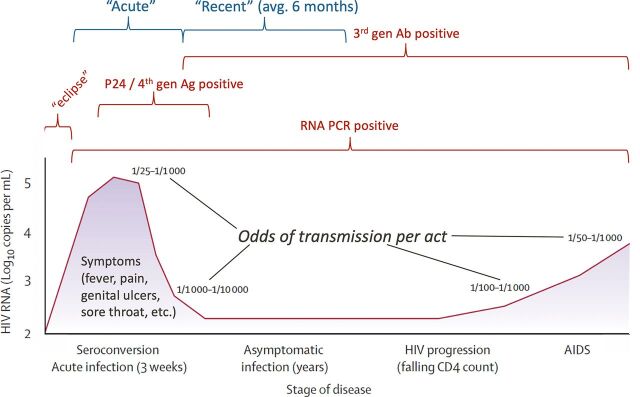
The Natural History of HIV Disease Progression in the Absence of Antiretroviral Therapy, Variable Transmission Probabilities Associated With Viral Burden, and Capacities for Detection of HIV Infection Through Different Diagnostics Abbreviations: Ag, antigen; Ab, antibody; PCR, polymerase chain reaction.

The brief duration of AHI poses a major challenge to diagnosis.^16^ ^,^ ^17^ Detection of AHI depends on infected individuals having a blood test during the short AHI period and then establishing the presence of HIV RNA or p24 antigen (part of the virus) (Figure 2). Individuals facing high infection risks would need to seek HIV testing with HIV RNA or p24 technologies on a frequent basis to increase the likelihood of detecting an infection during the acute period.

The brief duration of acute infection and cost of testing poses barriers to diagnosis.

Cost is also an issue. Point-of-care platforms for detection of HIV RNA such as Alere Q (Abbott Laboratories) and GeneXpert (Cepheid) are now available but are generally perceived as expensive (US$17–24). Fourth-generation point-of-care rapid HIV tests, such as the Alere HIV Combo kit (Abbott Laboratories), detect both p24 antigen and HIV antibodies within 20 minutes at a lower cost (US$2–4) and can be substituted as the first, sensitive screening test in a national HIV testing algorithm for diagnosis.^17^ However, these fourth-generation assays have much lower sensitivity to detect AHI than HIV RNA assays.^39^

Nevertheless, a clinical trial of PrEP in Uganda, South Africa, and Zimbabwe found that 28% of infections missed by current third-generation rapid diagnostic tests would have been identified with the use of Alere HIV Combo, suggesting some advantages of using a fourth-generation test over standard antibody testing.^40^ Investigators in San Francisco found more promising results, with the Alere Determine (Abbott Laboratories) point-of-care fourth-generation antigen-antibody combo rapid test detecting about 54% of the acute cases detected through laboratory RNA testing.^41^ In Thailand, the Alere HIV Combo kit detected 37 of 50 (74%) individuals with HIV RNA confirmed acute HIV infection. These limited results suggest promising performance of Alere HIV Combo in a facility-based setting but require broader evaluation in diverse settings and populations.^42^ ^,^ ^43^

AHI is sometimes accompanied by transient clinical flu-like and other symptoms, including rash, fever, sore throat, fatigue, muscle/joint aches, oral and genital ulcers, diarrhea, and swollen lymph nodes.^44^ ^,^ ^45^ However, these symptoms and signs are neither sensitive nor specific for AHI. Inquiring about the presence of these symptoms and recent risk behaviors may suggest opportunities to screen for AHI with an RNA assay if available or at least a fourth-generation test.^46^ ^–^ ^48^ Sensitizing populations at risk to AHI signs and symptoms, benefits of early detection and treatment, and potential advantages of fourth-generation diagnostics may also facilitate improved AHI diagnosis and treatment and mitigate HIV resistance risks associated with PrEP continuation among individuals who may have received false-negative third-generation HIV testing results.

To overcome some of the logistical and resource-related barriers to the expansion of AHI screening, diagnosis, and intervention, we propose 2 potential solutions to encourage further consideration of both these and other context-relevant approaches: (1) conducting pooled HIV-1 RNA polymerase chain reaction (PCR) on dried blood spot samples; and (2) expanding the use of fourth-generation point-of-care rapid HIV testing, leveraging recency testing data where possible to help focus AHI screening in networks in which ongoing HIV transmission is occurring.

## POOLED PCR TO FACILITATE DETECTION OF AND INTERVENTION DURING AHI

The “gold standard” for detection of AHI is molecular testing, specifically HIV-1 RNA PCR. This approach is considered the standard of care to facilitate early infant diagnosis among children born to HIV-infected mothers. However, PCR is relatively expensive.^17^ To extend PCR testing efficiently to all individuals facing elevated HIV infection risks but who have nonreactive serological testing results as part of a targeted HIV testing strategy, samples can be “pooled” such that qualitative PCR is run on a batch that combines like sample types sourced from different individuals. Individual results are confirmed as negative for negative pools. For reactive pools, each sample in the pool is then tested with quantitative PCR viral load independently to identify and rapidly intervene with individuals with reactive results. The Thai Red Cross AIDS Research Center has been applying a pooling approach with plasma samples as a cost-efficient strategy to identify and treat individuals with AHI who might otherwise not receive a diagnosis using serological testing.^49^ Nevertheless, separation, storing, and transfer of plasma can pose logistical challenges in resource-limited settings and incur additional costs. Pooled PCR testing may also be possible on point-of-care viral load platforms as these become more affordable and widely available, realizing additional benefits in terms of efficiency, convenience, and early detection and intervention.

**FIGURE 3.**
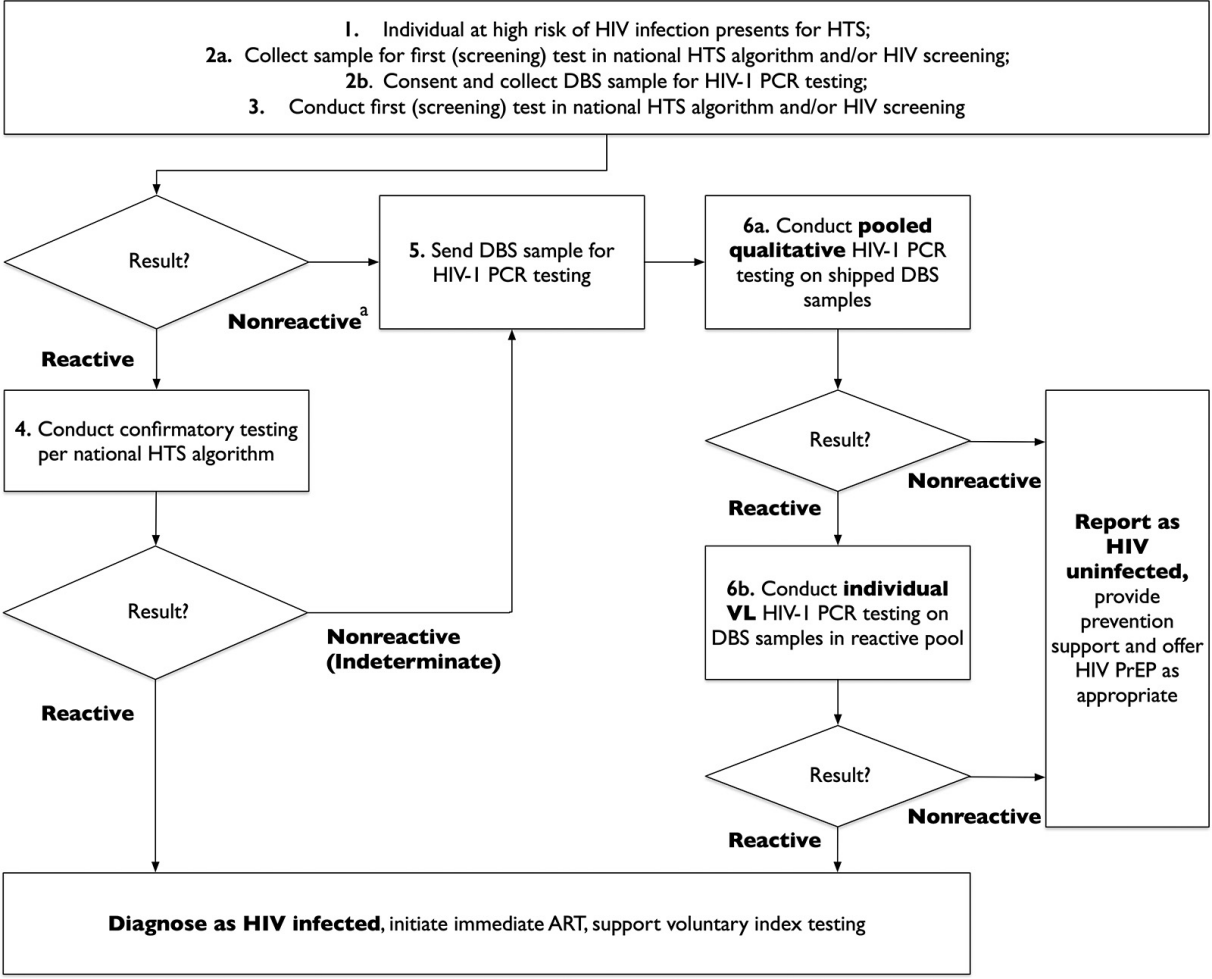
An Algorithm for Routine Screening for Acute HIV Infection in Populations Facing Elevated HIV Infection Risks Abbreviations: ART, antiretroviral therapy; DBS, dried blood spot; HTS, HIV testing services; PCR, polymerase chain reaction; PrEP, pre-exposure prophylaxis; VL, viral load.^a^ Individuals with nonreactive results on the first or “screening” test would be notified as likely uninfected but as falling within a window period for possible HIV infection pending the outcome of the DBS HIV PCR testing.

In resource-limited settings, collecting dried blood spot samples for pooled PCR is a promising approach to circumvent some of the cost barriers associated with point-of-care platforms and the logistical challenges associated with separation, storing, and transfer of plasma samples. Whole blood spots can be collected with a finger prick and can be stored and shipped with relative ease. Recalibration of the PCR is necessary because of a degradation of viral RNA in dried blood that may result in a 2-log reduction in assay sensitivity and because of the potential presence of viral DNA, which may partially compensate for this loss in sensitivity.^50^ At least 1 study has demonstrated the accuracy of doing pooled PCR on dried blood spot samples for early infant diagnosis and documented a laboratory cost savings of 65% associated with pooling.^50^ Other studies have demonstrated feasibility to diagnose AHI and to reduce the costs of ART monitoring in resource-limited settings with pooled PCR on dried blood spot samples.^51^ ^,^ ^52^ An illustrative depiction of this diagnostic algorithm incorporating AHI screening based on dried blood spot sample pooled PCR testing is provided in Figure 3.

In resource-limited settings, collecting dried blood spot samples for pooled PCR is a promising approach to circumvent some logistical and cost barriers to detecting acute HIV infection.

To maximize the benefits of screening for AHI, the time from sample collection to case identification and intervention must be minimized. A pooling approach is more practical in high volume settings in which batches can be run every day. While the time needed to process samples will vary according to the proximity and availability of laboratory infrastructure, we anticipate many programs being able to provide results in 1–2 days given the predominantly urban concentration of laboratory resources and of HIV key population risks in many country settings. The expansion of point-of-care viral load testing can further reduce turnaround times, facilitating earlier action.

The routine collection of dried blood spot samples to detect and intervene during AHI also presents some additional value-added opportunities to improve HIV service delivery that are worthy of consideration. Drug-resistance genotyping can be performed on samples from all individuals who are confirmed through either molecular or serological testing to have HIV infection. In addition, these samples could be used to conduct phylogenetic analyses of potential HIV transmission clusters to enhance the focus of targeted testing and index testing implementation.^53^

## COMBINING AHI SCREENING WITH RECENCY TESTING TO FACILITATE EARLIER HIV DIAGNOSIS AND INTERVENTION

Rapid HIV recency assays, such as the Asanté HIV-1 Rapid Recency® Assay (Sedia Biosciences) and the Maxim Swift HIV Recent Infection Assay (Maxim Biomedical Inc.), were developed to help identify individuals who have become HIV infected within the past year—on average in the past 6 months—to help estimate HIV incidence and improve the focus of programming in settings, populations, and networks in which incident infections continue to occur.^54^ Rapid recency point-of-care antibody-based assays differentiate between recent HIV infection—when the antibody response is immature, as reflected by low “avidity” or binding strength of the antibody—and long-term infections in which a mature antibody response is measured by strong antibody avidity.^55^ ^,^ ^56^ The assays can yield “false-recent” results among individuals who naturally control HIV well (low virus=low antibody) or are receiving ART, so a recent infection result is usually confirmed using a recent infection testing algorithm in which a viral load test is conducted with results of ≥ 1,000 copies/mL confirming recent infection.^54^ ^,^ ^57^ ^,^ ^58^

Rapid recency assays only measure antibody avidity after HIV seroconversion; they do not detect HIV RNA or p24 antigen and therefore are unable to detect AHI. In typical use, they are only offered to individuals who have been confirmed to have HIV infection with a standard antibody-based national HIV testing algorithm. Recency assays are also pending review for diagnostic purposes by the World Health Organization and are currently only approved for research use by the U.S. Food and Drug Administration. The World Health Organization has endorsed the use of recency assays for surveillance purposes but has not yet made a determination regarding program or individual-level benefits pending further evidence.^54^

That said, the rapid recency algorithm has demonstrated capacity to identify individuals who became HIV infected within the past year, and the U.S. President’s Emergency Plan for AIDS Relief has identified the scale up of recency testing as a “minimum standard” for HIV program implementation in an expanding set of countries receiving U.S. government support.^59^ While there currently is no rationale to offer differentiated counseling or clinical HIV treatment support to PLHIV with recent versus long-term HIV infection, it is more likely that persons with recent infections are part of ongoing transmission networks. Individuals with recent infection were recently acutely infected and were recently in risk contact with at least 1 other PLHIV who was not virally suppressed. Therefore, targeting testing among the contacts of recently infected individuals could improve the capacity of programs to detect and treat previously undiagnosed individuals while focusing prevention services among individuals facing the greatest infection risks. Moreover, conducting AHI screening among the network contacts of recently infected individuals, as well as targeting AHI screening among individuals with similar risk and sociodemographic profiles to those with recent infections, could increase capacity to detect, treat, and prevent transmission during AHI.

Testing the contacts of recently infected individuals could improve a program’s capacity to detect and treat undiagnosed individuals.

To leverage recency testing data to help focus AHI screening as part of an HIV micro-epidemic control model, programs would need to adopt strategies to: (1) integrate AHI screening into practice; (2) secure client informed consent for recency testing and the confidential use of those results; (3) support confidential profiling of individuals with recent and acute infections; (4) target testing with AHI screening in populations, settings, and networks aligned to these profiles; and (5) prioritize index testing with AHI screening among the contacts of recently and acutely infected individuals.

**FIGURE 4.**
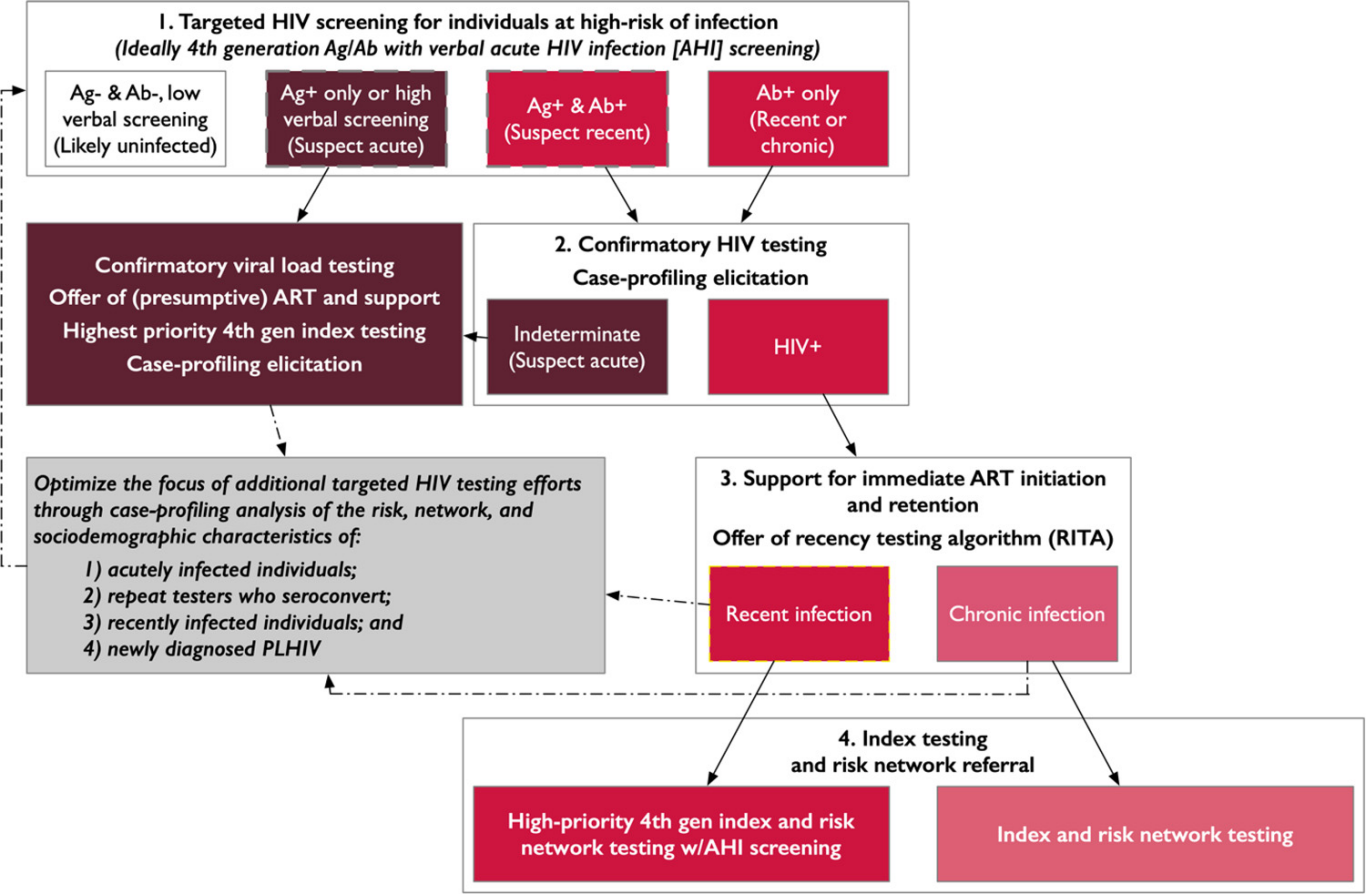
An Illustrative Workflow for Acute HIV Infection Screening, Additionally Applying Recency Testing Data to Help Improve Focus Abbreviations: Ab+, antibody positive; Ab−, antibody negative; AHI, acute HIV infection; Ag+, antigen positive; Ag−, antigen negative; ART, antiretroviral therapy; HIV+, HIV-positive; PLHIV, people living with HIV.

An illustrative workflow for AHI screening supplemented by recency testing to improve focus is shown in Figure 4. AHI screening could be conducted using a pooled PCR approach on dried blood spot samples as previously described. However, in this instance, we outline an approach in which members of key and priority populations could be offered screening for AHI through combined use of a sensitive fourth-generation rapid diagnostic test like the Alere HIV Combo, as well as a risk- and symptom-based verbal screening tool. A potentially useful example of a tool validated using AHI data from the Amsterdam Cohort Studies among men who have sex with men consisted of a self-administered weighted survey inquiring about the presence of 4 current symptoms (fever, lymphadenopathy, oral thrush, and weight loss), and 3 risk factors in the past 6 months (receptive condomless anal intercourse, more than 5 sexual partners, and gonorrhea).^47^

Prioritizing AHI screening as part of index testing for the risk network contacts of recently infected individuals, as well as eventually for the contacts of individuals with AHI as these are identified, could then help programs increase the likelihood of detecting people with AHI. Case profiles can be developed to describe actionable characteristics of recently and acutely infected individuals to guide the prioritization of targeted, differentiated HIV testing, AHI screening, and prevention strategies for these individuals and members of their risk networks. Individuals who are recently or acutely infected can also be offered opportunities to serve as peer mobilizers and/or to distribute HIV self-test kits to help accelerate linkages of their network contacts to testing, treatment, and prevention services, as relevant.

As individuals are screened as potentially having AHI, these individuals can be offered immediate confirmatory HIV RNA testing and HIV treatment. On the rare occasion that HIV RNA confirmatory testing is not immediately available, presumptive HIV treatment could be provided pending confirmation in a manner analogous to the provision of HIV postexposure prophylaxis, which is generally considered safe and effective.^60^ An advantage of the proposed pooled PCR approach to detect AHI is immediate confirmation of HIV infection. Once confirmation is obtained, individuals can be sustained on ART, having gained personal immunological benefits from early treatment and having reduced the likelihood of ongoing HIV transmission during AHI. For individuals who were screened as having presumptive AHI but who later are determined through HIV RNA testing to be uninfected, treatment can be discontinued with minimal risk of harm or of contributing to development of drug-resistant HIV, in a fashion similar to the discontinuation of HIV postexposure prophylaxis. These persons can also be assessed for the suitability of PrEP.

Current concerns about the potential impact of providing acute or recency test results to clients include increased risk of criminalization of key populations, as well as criminalization of HIV transmission and increased risk of gender-based or intimate partner violence.^61^ Furthermore, subjecting patients to tests like recency assays that do not provide additional clinical benefits raises ethical concerns. In principle, patients have a right to know any information that is part of their medical file, and additional information about the current state of a person’s infection may help providers enhance counseling, reinforce a person’s reduction in risk behaviors that lead to onward transmission, improve partner elicitation process within index testing services, and allow providers to use results to prioritize index cases for partner notification services. Adverse events or harm related to return of acute or recency results have not been reported so far from early programs implementing these services,^54^ ^,^ ^62^ ^,^ ^63^ but few studies have systematically evaluated outcomes related to potential harm, client perspectives, or the perspectives of partners of index clients. Given the potential public health benefit of engaging the risk contacts of recently or acutely infected individuals, the assessment of these outcomes is imperative to provide guidance around the messaging of results in a manner which minimizes risks and optimizes potential benefits.

## CONCLUSIONS

Viral burden is the primary predictor of HIV-related morbidity, mortality, and ongoing transmission. Although a majority of PLHIV globally have achieved viral suppression through sustained access to HIV treatment, achieving an end to the HIV pandemic is contingent on addressing the preferences and needs of virally unsuppressed and persistently unserved and underserved PLHIV and members of their risk networks. Proven solutions exist to prevent and treat HIV, but the approaches and technologies needed to differentiate and focus support based on viral burden historically have been limited. Now, with the expansion of viral load testing and an expanded set of options to screen for and treat AHI, we may be better equipped to improve both the impact and efficiency of efforts to accelerate epidemic control.

An HIV micro-epidemic control approach that prioritizes personalized treatment support for PLHIV who are not virally suppressed—and in the process focuses HIV testing and relevant HIV prevention and treatment support among their network members—offers a framework to integrate these advances into current practice to maximize client benefits and overall impact. In particular, such an approach offers a path to integrate the detection and treatment of AHI into routine programming, potentially curbing a substantial proportion of ongoing HIV transmission that occurs during this period and has historically continued apace beyond the reach of efforts to leverage HIV treatment as prevention at scale. However, the ultimate advantages of such an approach remain largely undocumented. Additional investments in the development, implementation, and evaluation of practical strategies to differentiate support based on viral burden are needed to assess the real-world benefit of the proposed HIV micro-epidemic control approach.

## Notes

**Peer Reviewed**

**First published online:** November 17, 2020
